# Mutations in the non-catalytic polyproline motif destabilize TREX1 and amplify cGAS-STING signaling

**DOI:** 10.1093/hmg/ddae089

**Published:** 2024-09-03

**Authors:** Abraham Shim, Xiaohan Luan, Wen Zhou, Yanick J Crow, John Maciejowski

**Affiliations:** 1 Molecular Biology Program, Sloan Kettering Institute, Memorial Sloan Kettering Cancer Center, New York, NY 10065, USA; 2 Department of Immunology and Microbiology, School of Life Sciences, Southern University of Science and Technology, Shenzhen, Guangdong 518055, China; 3 MRC Human Genetics Unit, Institute of Genetics and Cancer, The University of Edinburgh, Edinburgh, UK; 4 Laboratory of Neurogenetics and Neuroinflammation, Imagine Institute, INSERM UMR1163, University Paris Cité, Paris, France

## Abstract

The cGAS-STING pathway detects cytosolic DNA and activates a signaling cascade that results in a type I interferon (IFN) response. The endoplasmic reticulum (ER)-associated exonuclease TREX1 suppresses cGAS-STING by eliminating DNA from the cytosol. Mutations that compromise TREX1 function are linked to autoinflammatory disorders, including systemic lupus erythematosus (SLE) and Aicardi-Goutières syndrome (AGS). Despite key roles in regulating cGAS-STING and suppressing excessive inflammation, the impact of many disease-associated *TREX1* mutations - particularly those outside of the core catalytic domains - remains poorly understood. Here, we characterize a recessive AGS-linked TREX1 P61Q mutation occurring within the poorly characterized polyproline helix (PPII) motif. In keeping with its position outside of the catalytic core or ER targeting motifs, neither the P61Q mutation, nor aggregate proline-to-alanine PPII mutation, disrupt TREX1 exonuclease activity, subcellular localization, or cGAS-STING regulation in overexpression systems. Introducing targeted mutations into the endogenous *TREX1* locus revealed that PPII mutations destabilize the protein, resulting in impaired exonuclease activity and unrestrained cGAS-STING activation. Overall, these results demonstrate that TREX1 PPII mutations, including P61Q, impair proper immune regulation and lead to autoimmune disease through TREX1 destabilization.

## Introduction

Type I interferonopathies, such as the monogenic disease Aicardi-Goutières syndrome (AGS), often involve chronic systemic and neurological autoinflammation and high levels of type I interferon (IFN) activity in the blood and cerebrospinal fluid (1). AGS can result from loss-of-function (or specific dominant-negative) mutations in *TREX1, RNASEH2A, RNASEH2B, RNASEH2C, SAMHD1*, and *ADAR1*, gain-of-function mutations in *IFIH1* (2,3). Mutations in *TREX1* are among the most common in AGS, accounting for nearly one-quarter of all AGS-linked mutations (4,5).

TREX1 is a 3′→5′ exonuclease that degrades cytosolic DNA to act as a nucleolytic antagonist of the cGAS-STING pathway (6–11). Binding to cytosolic DNA stimulates cGAS catalytic activity and the production of the 2′3′-cyclic GMP-AMP (cGAMP) second messenger (12–14). cGAMP engagement with its downstream receptor STING ultimately results in activation of the transcription factor IRF3 and the expression of type I IFNs and other immunomodulatory proteins (15).

Mouse models of TREX1 dysfunction recapitulate hallmarks of AGS and related disorders, including familial chilblain lupus. *Trex1*-deficient mice exhibit multi-organ inflammation and decreased survival (8,11). Replacement of the wild-type *Trex1* gene in mice with the nuclease-deficient *Trex1* D18N mutant results in a lupus-like disease (8). The health and viability of *Trex1*-deficient animals are restored by deletion of *Cgas, Sting1, Irf3* and *Ifnar*, indicating that unchecked DNA sensing is responsible for the observed pathologies (6,9,11,16,17).

Specific dominant-negative mutations in *TREX1* include D18N, D200N, and H195Y, which disrupt key catalytic residues, and more frequently observed recessive alleles, e.g. R114H and R97H, that occur in the dimerization surface and hinder requisite homodimerization of TREX1 (2,18). Other less-common mutations have been proposed to impede TREX1 function by altering phase separation or by destabilizing the protein (19,20). The mechanisms associated with many disease-linked *TREX1* mutations are poorly understood.

Outside of its catalytic core, TREX1 possesses a single-pass transmembrane helix at its C-terminus that anchors the protein in the ER and positions the nuclease domain in the cytosol (7,10,21,22). Deleting this C-terminal extension ablates TREX1 ER localization but does not affect its catalytic activity (21,23). *TREX1* mutations that truncate the C-terminus disrupt TREX1-ER association while preserving nucleolytic activity, and are associated with a distinct clinical disease referred to as retinal vasculopathy with cerebral leukoencephalopathy (RVCL) (24,25). RVCL is inherited in an autosomal dominant manner and lacks clear links to excessive type I IFN production (26).

The non-repetitive proline-rich region termed the polyproline II helix (PPII) is another unique motif present in TREX1, but not found in other nucleases within the larger DnaQ family, including the closely related TREX2 homolog (23,27). Like the TREX1 C-terminal extension, the positioning of the PPII helix distal to the TREX1 active site and its absence from the otherwise closely related, catalytically active TREX2 nuclease suggest that it is also unlikely to participate in catalysis or DNA binding. The functional significance of this domain is not known.

Here, we report that TREX1 P61Q mutations located in the PPII motif are linked with AGS and show how these mutations destabilize TREX1 without directly affecting nucleolytic activity or subcellular localization. We demonstrate that TREX1 P61Q instability causes overactive cGAS-STING signaling, ultimately resulting in cGAMP overproduction and excessive levels of type I IFN expression. Thus, these results indicate that the TREX1 P61Q mutations cause AGS through TREX1 protein destabilization and suggest that protein destabilization may account for a subset of AGS patients with *TREX1* mutations.

## Results

### TREX1 PPII mutations are associated with AGS

**Figure 1:**
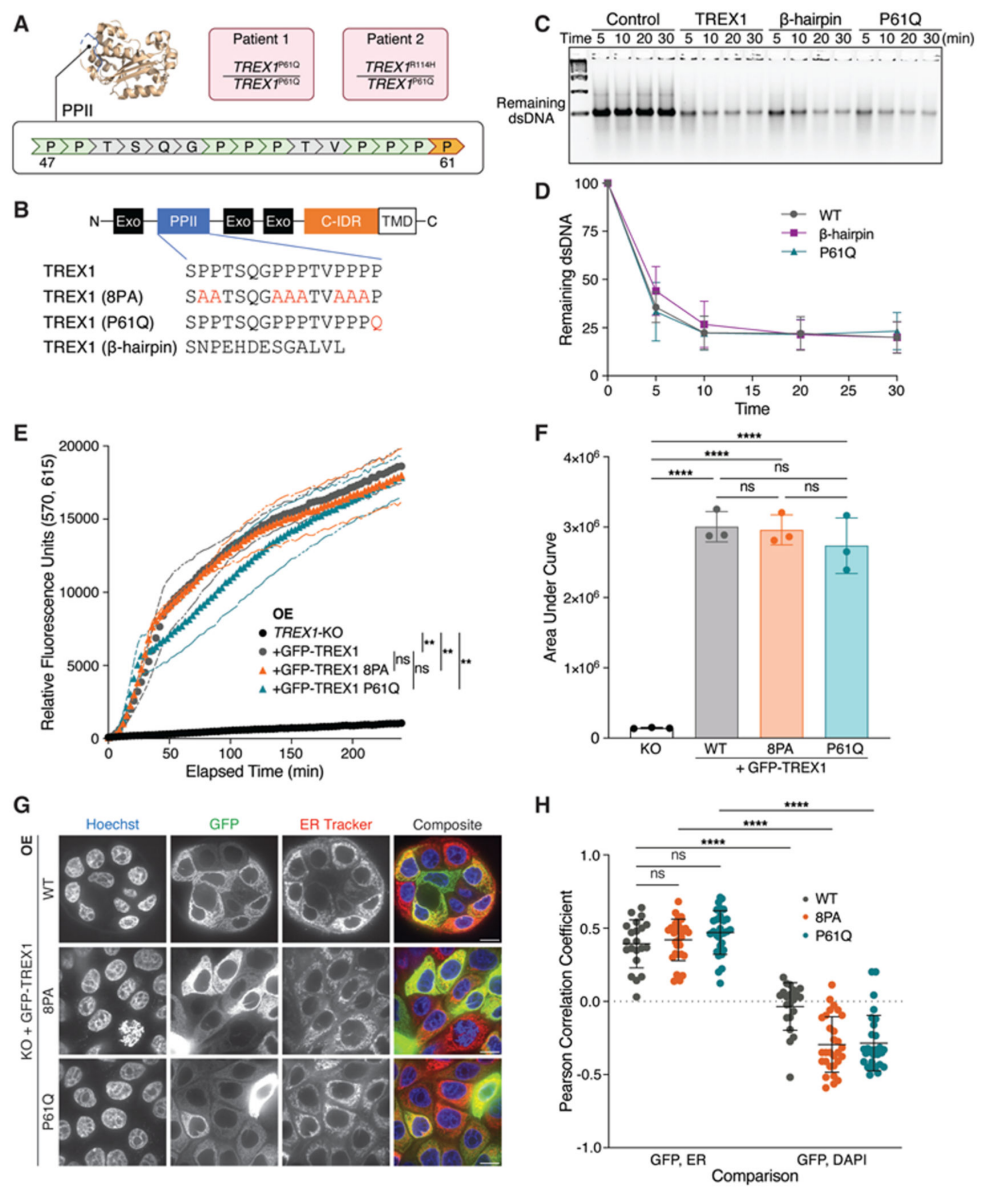
Mutations in PPII are linked to AGS but do not compromise intrinsic functions of overexpressed TREX1. **A**. Location of PPII and P61 (orange) within TREX1. Genotypes of two AGS patients harboring the P61Q mutation are shown in pink. **B**. Schematic of GFP-TREX1 mutants used to reconstitute MCF10A *TREX1* KO cells via lentiviral overexpression. Exo = exonuclease domain; C-IDR = C-terminal intrinsically disordered region; TMD = transmembrane domain responsible for TREX1-ER linkage. **C**. Representative DNA gel from *in vitro* nuclease assay. A dsDNA substrate was co-incubated with purified TREX1 mutant protein for the indicated duration. Control = no TREX1 added; β-hairpin = TREX1 with PPII replaced with TREX2 β-hairpin occurring at corresponding position as TREX1. P61Q = TREX1 P61Q **D**. Quantification of the *in vitro* nuclease assay in (C); mean ± s.d., *n* = 3, two-way ANOVA (interaction *p* = 0.5020). **E**. Time course fluorescence reading of the lysate-based nuclease assay. Briefly, a dsDNA substrate labeled with adjacent TEX615 fluorophore and Iowa Black quencher was co-incubated with whole cell lysates. 3′→5′ exonuclease activity eliminates the quencher, liberating TEX615 fluorescence; mean ± s.d., *n* = 3, **p < 0.01, ns = not significant, two-way ANOVA (interaction *p* < 0.0001, time *p* < 0.0001, genotype *p* < 0.0001). **F**. Definite integral values from *t* = 0 min to *t* = 240 min for each time course sample in (E); mean ± s.d., *n* = 3, ****p < 0.0001, ns = not significant, one-way ANOVA (*p* < 0.0001). **G**. Live-cell images of GFP-TREX1 (green) in *TREX1*-KO cells. DNA was stained with Hoechst 33342 (blue) and ER was stained with ER Tracker Red (red). Scale bars = 10 μm. **H**. Pearson correlation coefficients of the indicated cells as in Fig. 1G; mean ± s.d., *n* = 5 experiments, ****p < 0.0001, ns = not significant, two-way ANOVA (interactions *p* < 0.0001, comparison pair *p* < 0.0001, genotype *p* = 0.0027). **Alt text: A**. Schematic of TREX1 protein crystal structure highlighting the amino acid sequence of the PPII motif. Genotypes of the two AGS patients harboring the P61Q mutation are shown. **B**. Linear schematic of TREX1 domains, with a callout highlighting the residues mutated in this study. **C**. DNA gel showing the remaining double-stranded DNA across four timepoints (5, 10, 20, and 30 minutes) for four genotypes (control, wildtype TREX1, beta-hairpin, and P61Q). **D**. Line graph depicting a decrease in the remaining double-stranded DNA as a function of time, for three genotypes: wildtype, beta-hairpin, and P61Q. **E**. Line graph showing an increase in relative fluorescence units as a function of time, for four genotypes: TREX1-knockout, GFP-TREX1-wildtype, GFP-TREX1-8PA, and GFP-TREX1-P61Q. **F**. Bar graph comparing area-under-curve values for four genotypes: TREX1-knockout, GFP-TREX1-wildtype, GFP-TREX1-8PA, and GFP-TREX1-P61Q. **G**. Panel of microscope images showing Hoechst, GFP, ER Tracker, and composite channels across three genotypes: GFP-TREX1-wildtype, GFP-TREX1-8PA, and GFP-TREX1-P61Q. **H**. Bar graph comparing Pearson correlation coefficient values between GFP and ER channels and between GFP and DAPI channels, across three genotypes: GFP-TREX1-wildtype, GFP-TREX1-8PA, and GFP-TREX1-P61Q.

We identified proline-to-glutamine (P61Q) point mutations in *TREX1* in two patients from two families presenting with features of AGS (Fig. 1A; AGS972: c.182C>A p.Pro61Gln homozygote; AGS1583: p.Pro61Gln/Arg114His compound heterozygote) (28). Since R114H renders the allele null by disrupting obligate dimerization of TREX1 (2), these findings suggest a recessive, loss-of-function nature of the P61Q mutation. Indeed, calculation of IFN scores, derived by measuring the expression of six IFN stimulated genes (ISGs) using quantitative polymerase chain reaction, revealed a significant upregulation of IFN signaling relative to persons considered to be controls, thus placing both individuals within the type I interferonopathy spectrum (25,28).

Pro-61 lies in a proline-rich tract termed the polyproline II (PPII) helix (Fig. 1A,B) (23,27). PPII positioning distal to the TREX1 active site and its absence from other catalytically proficient enzymes of the DnaQ family, including TREX2, suggest it is likely to be dispensable for nucleolytic activity. To test this directly, we purified the N-terminal enzymatic domain of TREX1 proteins, including human TREX1, a TREX1 P61Q mutant, and a TREX1 PPII>β-hairpin chimera, in which the TREX1 PPII helix is replaced by the β-hairpin found in the corresponding position within TREX2 (Fig. 1B). As expected, *in vitro* nuclease assays using purified proteins demonstrated that TREX1 P61Q and β-hairpin mutants digested dsDNA with efficiencies comparable to the wild-type enzyme with >50% of substrate degraded within the first 5 minutes of incubation (Fig. 1C,D).

To further investigate the potential impact of TREX1 PPII mutations we assayed TREX1 exonuclease activity in cell lysates. In brief, lysates were incubated with a dsDNA substrate possessing a fluorescent label at one 5′ end closely positioned next to a 3′ quencher (Methods). TREX1 3′→5′ exonuclease activity is predicted to liberate the fluorescent dye from the 3′ quencher and thus result in the acquisition of fluorescence. Cell lysates were prepared from *TREX1*-deficient MCF10A cells stably transduced with GFP-TREX1-WT, GFP-TREX1-P61Q, and GFP-TREX1-8PA, in which eight prolines in PPII - excluding P61 - are mutated to alanine (Fig. 1B). Lentiviral transduction of these constructs into *TREX1*-deficient MCF10A cells yielded stable overexpression of GFP-tagged mutant proteins, with no significant differences in protein levels between the three genotypes (Fig. S1A and S1B). As expected, incubation of the dsDNA probe with lysates prepared from MCF10A *TREX1* KO cells reconstituted with GFP-TREX1-WT resulted in the rapid acquisition of fluorescence (Fig. 1E,F). In contrast, *TREX1* deletion severely diminished the acquisition of fluorescence, confirming the specificity of this assay for TREX1 exonuclease activity (Fig. 1E,F). Similar to results obtained using isolated proteins, measurement of GFP-TREX1-8PA and GFP-TREX1-P61Q activities exhibited no significant differences from GFP-TREX1-WT (Fig. 1E,F). Taken together, these data indicate that targeted mutations within the PPII helix do not directly interfere with TREX1 exonuclease activity and suggest that the PPII helix is dispensable for TREX1 exonuclease activity against dsDNA.

We previously demonstrated that TREX1 association with the ER is critical for processing a subset of cytosolic DNA substrates including nuclear aberrations like micronuclei (22). Positioning of the PPII within the catalytic core and distal to the ER transmembrane domain at the C-terminus of TREX1 suggested that the PPII domain is likely dispensable for ER association. To test this possibility directly, we performed live-cell imaging of cells overexpressing GFP-TREX1 mutants to characterize their subcellular localization. As previously reported (10,11,22), GFP-TREX1-WT was excluded from the nucleus and its localization significantly overlapped with the ER, as indicated by staining with an ER tracker dye (Fig. 1G,H). GFP-TREX1-8PA and GFP-TREX1-P61Q subcellular localizations could not be distinguished from that of the wild-type enzyme, suggesting that the PPII is dispensable for directing TREX1 ER association (Fig. 1G,H; Figure S1C,D). Together, these data indicate that PPII mutations are unlikely to cause TREX1 dysfunction by interfering with its ER localization.

### Overexpressed TREX1 PPII mutants suppress cGAS-STING signaling

**Figure 2:**
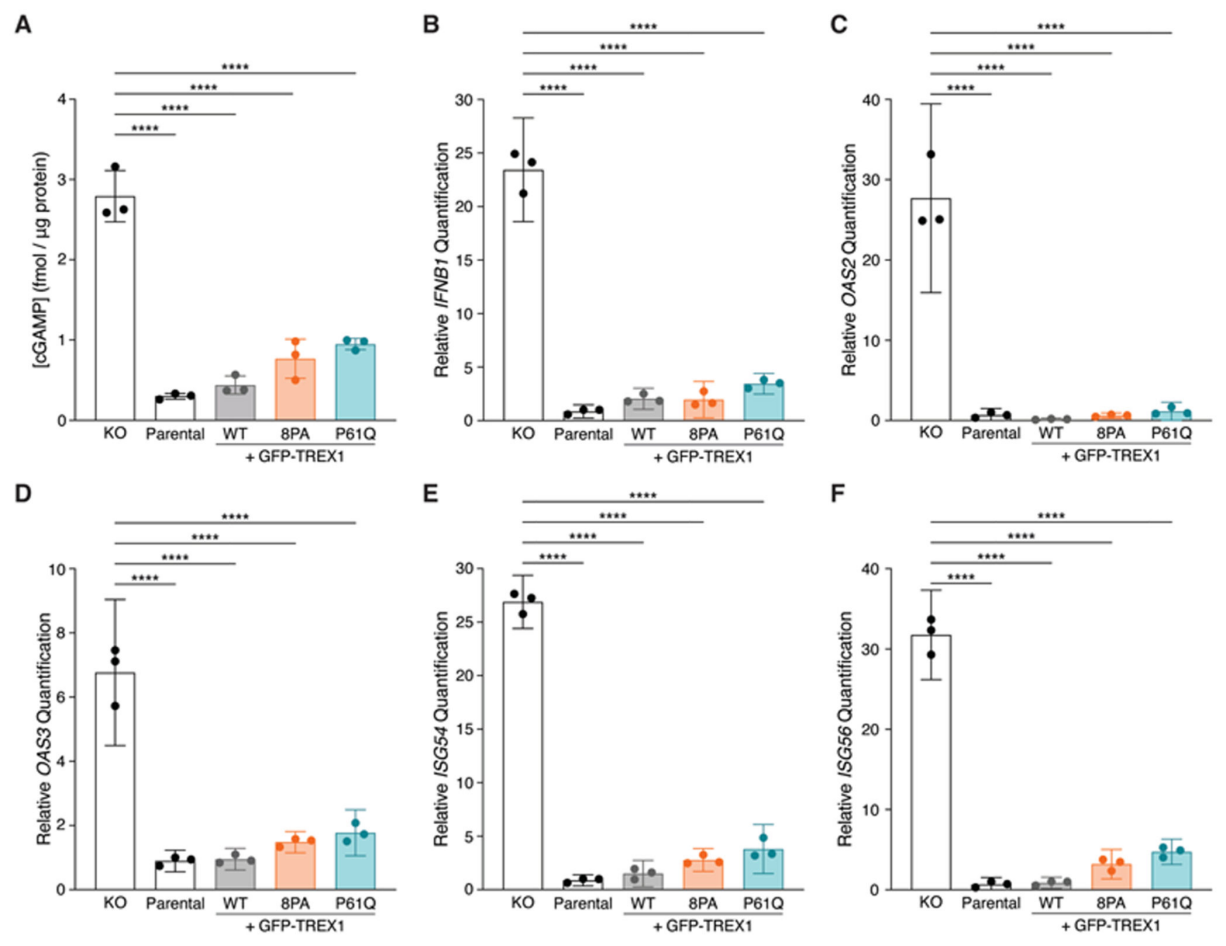
Overexpressed TREX1 mutants can suppress cGAS-STING signaling. **A**. ELISA analysis of cGAMP production in the indicated cells following the transfection of 4 μg HT-DNA; mean ± s.d., *n* = 3, ****p < 0.0001, one-way ANOVA (*p* < 0.0001). **B–F**. RT-qPCR of *IFNB1, OAS2, OAS3, ISG54*, and *ISG56* expression in the indicated MCF10A cells following the transfection of 4 μg HT-DNA; mean ± s.d., *n* = 3, ****p < 0.0001, one-way ANOVA (*p* < 0.0001 for *IFNB1, p* < 0.0001 for *OAS2, p* < 0.0001 for *OAS3, p* < 0.0001 for *ISG54*, and *p* < 0.0001 for *ISG56*). **Alt text: A**. Bar graph comparing cGAMP concentration values across five genotypes: TREX1-knockout, parental, GFP-TREX1-wildtype, GFP-TREX1-8PA, and GFP-TREX1-P61Q. **B–F**. Bar graphs comparing mRNA relative quantification values across five genotypes: TREX1-knockout, parental, GFP-TREX1-wildtype, GFP-TREX1-8PA, and GFP-TREX1-P61Q. Genes quantified: IFNB1 for B, OAS2 for C, OAS3 for D, ISG54 for E, and ISG56 for F.

To test whether PPII mutations affect cGAS activation, we quantified intracellular cGAMP via ELISA (Fig. 2A). MCF10A cells lack high levels of cytosolic DNA and do not show strong cGAS activity at baseline, even upon *TREX1* deletion (19,22). We therefore stimulated cGAS activation by herring testes (HT-) DNA transfection. ELISA analysis revealed low to undetectable amounts of cGAMP (0.3005±0.03630 s.d. fmol/μg protein) in MCF10A cells after HT-DNA stimulation (Fig. 2A). As expected, cGAMP levels increased dramatically in *TREX1* KO cell lysates following HT-DNA transfection (2.792±0.3207 s.d. fmol/μg protein) (Fig. 2A). Reconstitution of MCF10A *TREX1* KO cells by overexpressing GFP-TREX1-WT diminished cGAMP to levels observed in the parental cell line (0.4398±0.1119 s.d. fmol/μg protein) (Fig. 2A). In keeping with their catalytic proficiency and normal ER localization, GFP-TREX1-8PA and GFP-TREX1-P61Q overexpression led to cGAMP reductions that were comparable to the wild-type GFP-TREX1 transgene (0.7678±0.2449 s.d. fmol/μg protein for GFP-TREX1-8PA; 0.9510±0.06908 s.d. fmol/μg protein for GFP-TREX1-P61Q).

We next sought to determine if TREX1 PPII mutations impacted the downstream cGAS-STING response by using RT-qPCR to measure expression of *IFNB1* and interferon-stimulated genes (ISGs) such as *OAS2, OAS3, ISG54*, and *ISG56* (Fig. 2B-F). As expected, RT-qPCR revealed strong increases in *IFNB1* and ISG mRNA levels in *TREX1* KO MCF10A cells upon HT-DNA stimulation relative to parental controls (Fig. 2B-F). In line with our cGAMP ELISA results, GFP-TREX1-WT, GFP-TREX1-8PA and GFP-TREX1-P61Q suppressed *IFNB1* and ISG expression to similar degrees upon overexpression in *TREX1* KO cells (Fig. 2B-F). Thus, counter to expectations based on the association between the TREX1 P61Q mutations and AGS (Fig. 1A), these results indicate that TREX1 PPII mutants are functionally proficient to suppress cGAS activation and downstream ISG expression upon overexpression in MCF10A cells.

### TREX1 PPII mutations destabilize the protein

**Figure 3:**
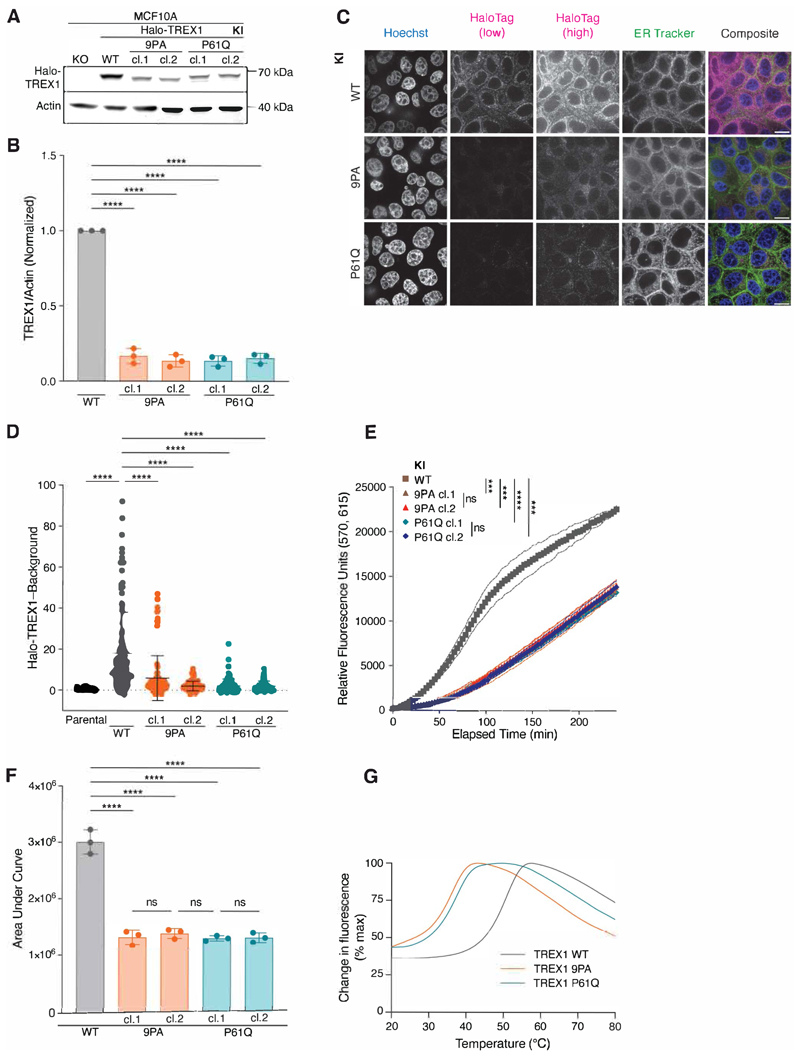
PPII mutations destabilize TREX1 and reduce TREX1 exonucleolytic activity. **A**. Immunoblot of MCF10A knock-in (KI) cell lines using anti-TREX1 and anti-actin antibodies. **B**. Quantification of TREX1 immunoblot signal normalized to actin; mean ± s.d., *n* = 3, ****p < 0.0001, one-way ANOVA (*p* < 0.0001). For each replicate, the WT TREX1/Actin signal was set to one. **C**. Live-cell images Halo-TREX1 (magenta) in MCF10A knock-in cell lines. DNA was stained with Hoechst 33342 (blue) and ER was stained with ER Tracker Green (green). Halo-TREX1 images are shown using two lookup tables in order to highlight differences in fluorescence signal (HaloTag low) and to depict ER localization (HaloTag high). Scale bars = 10 μm. **D**. Quantification of Halo-TREX1 signal in the indicated MCF10A cells as in (E); mean ± s.d., *n* = 5 experiments, ****p < 0.0001, one-way ANOVA (*p* < 0.0001). **E**. Time course fluorescence reading of lysate-based nuclease assay, using MCF10A knock-in cell lines; mean ± s.d., *n* = 3, ***p < 0.001, ****p < 0.0001, ns = not significant, two-way ANOVA (interaction *p* < 0.0001, time *p* < 0.0001, genotype *p* < 0.0001). **F**. Definite integral values from *t* = 0 min to *t* = 240 min for each time course sample in (E); mean ± s.d., *n* = 3, ****p < 0.0001, one-way ANOVA (*p* < 0.0001). **G**. Thermal shift assay using purified TREX1 proteins. T_m_ = 51 ºC for TREX1-WT, T_m_ = 36.5 ºC for TREX1-9PA, T_m_ = 37.5 ºC for TREX1-P61Q. **Alt text: A**. Western blot showing bands for Halo-TREX1 (about 70 kilodaltons) and actin (40 kilodaltons) across six samples: TREX1-knockout, Halo-TREX1-wildtype, two clones of Halo-TREX1-9PA, and two clones of Halo-TREX1-P61Q. **B**. Bar graph comparing TREX1-to-actin ratio values across five samples: Halo-TREX1-wildtype, two clones of Halo-TREX1-9PA, and two clones of Halo-TREX1-P61Q. **C**. Panel of microscope images showing Hoechst, HaloTag (two exposures), ER Tracker, and composite channels across three genotypes: Halo-TREX1-wildtype, Halo-TREX1-9PA, and Halo-TREX1-P61Q. **D**. Bar graph comparing Halo-TREX1 intensity values minus background across six samples: parental, Halo-TREX1-wildtype, two clones of Halo-TREX1-9PA, and two clones of Halo-TREX1-P61Q. **E**. Line graph showing an increase in relative fluorescence units as a function of time, for five samples: Halo-TREX1-wildtype, two clones of Halo-TREX1-9PA, and two clones of Halo-TREX1-P61Q. **F**. Bar graph comparing area-under-curve values for five samples: Halo-TREX1-wildtype, two clones of Halo-TREX1-9PA, and two clones of Halo-TREX1-P61Q. **G**. Line graph showing change in fluorescence as a percentage of maximal value, across temperature, for three genotypes of TREX1: wildtype, 9PA, and P61Q.

We reasoned that strong TREX1 overexpression resulting from lentiviral delivery (Fig. S1A and S1B) may obscure defects associated with PPII mutation. We therefore used CRISPR-Cas9 gene editing to endogenously introduce an N-terminal HaloTag concurrently with a PPII edit—proline-to-alanine mutation of all nine prolines in PPII (9PA) or P61Q - into the diploid MCF10A cell line (Fig. S2A). The remaining, unedited allele was deleted, yielding Halo-TREX1/Δ genotypes for all subsequent experiments (Fig. S2A). All gene edits were validated by Sanger sequencing and PCR screening (Fig. S2B–D). Immunoblotting with anti-TREX1 antibodies further confirmed successful insertion of the HaloTag into the endogenous *TREX1* locus. (Fig. 3A).

Interestingly, immunoblotting revealed significantly diminished Halo-TREX1-9PA and Halo-TREX1-P61Q signals in multiple, independently isolated subclones relative to the wild-type Halo-TREX1 control (Fig. 3A,B). Live-cell imaging confirmed decreased expression of Halo-TREX1-9PA and Halo-TREX1-P61Q relative to wild-type Halo-TREX1 (Fig. 3C and 3D). As expected, neither mutation compromised the ER localization of TREX1 (Fig. S3A and S3B). Similar to our prior results from GFP-TREX1 overexpression (Fig. 1E and 1F), all Halo-TREX1 lysates retained the ability to digest dsDNA (Fig. 3E and 3F). However, fluorescence increased at a much slower rate in Halo-TREX1-9PA/Δ and Halo-TREX1-P61Q/Δ lysates than Halo-TREX1-wild-type/Δ lysates, with the area under curve values decreased about two-fold. Thus, TREX1-P61Q and TREX1-9PA mutations lead to significant reductions in protein levels that are associated with corresponding decreases in nucleolytic activity.

Observed reductions in TREX1-9PA and TREX1-P61Q protein levels and activity could not be explained by reduced *TREX1* mRNA expression (Fig. S3C). Instead, Thermofluor analysis of purified proteins demonstrated significant differences in protein stability, with melting temperature (T_m_) values of 51 ºC for TREX1-WT, 36.5 ºC for TREX1-9PA, and 37.5 ºC for TREX1-P61Q (Fig. 3G and S3D). This destabilizing effect of the mutations was also observed in cells, as indicated by a cycloheximide (CHX) chase assay (Fig. S3E and S3F). Upon CHX-mediated inhibition of translation, TREX1-WT protein depleted to about 70–80 % of its initial level by 24 hours. On the other hand, TREX1-9PA and TREX1-P61Q levels declined more rapidly plateauing at about 40 % and 20 % of their initial levels, respectively. Overall, these results show that TREX1 PPII mutations destabilize the protein, and thus lead to reduced overall protein levels with corresponding decreases in nucleolytic activity.

### cGAS-STING signaling is elevated in TREX1 PPII mutant cells

**Figure 4:**
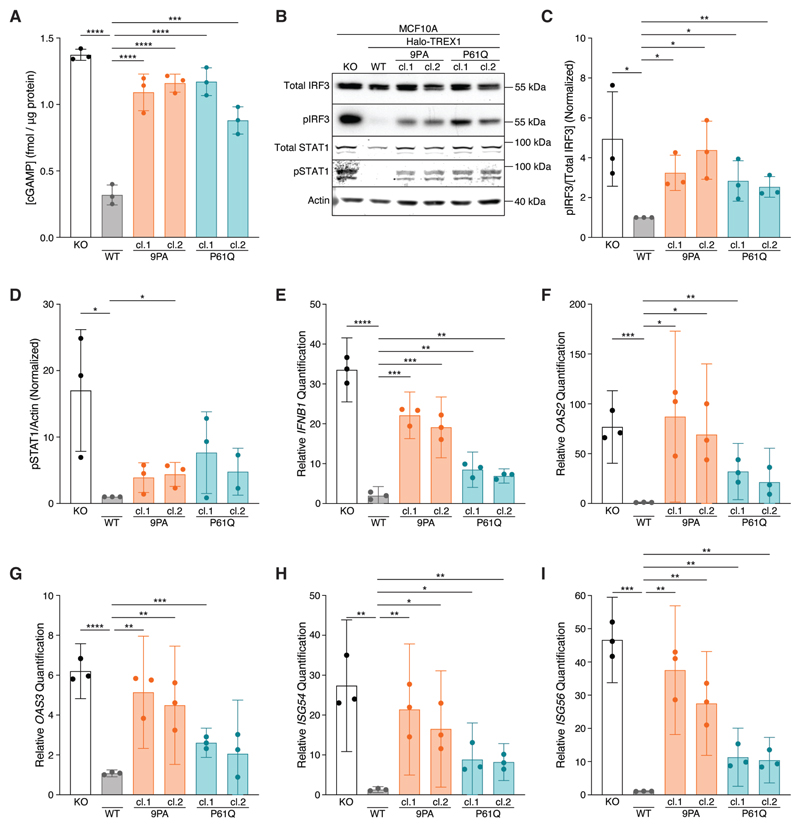
Mutations in PPII activate cGAS-STING signaling. **A**. ELISA analysis of cGAMP production in the indicated cells following the transfection of 4 μg HT-DNA; mean ± s.d., *n* = 3, ***p < 0.001, ****p < 0.0001, one-way ANOVA with post-hoc pairwise comparisons (*p* < 0.0001). **B**. Immunoblot of MCF10A knock-in (KI) cell lines using anti-IRF3, anti-phospho-S386 IRF3, anti-STAT1, anti-phospho-Y701 STAT1, and anti-actin antibodies. **C**. Quantification of phospho-S386 IRF3 immunoblot signal normalized to total IRF3 as in Fig. 4B; mean ± s.d., *n* = 3, *p < 0.05, **p < 0.01, unpaired two-tailed *t*-tests. For each replicate, the WT pIRF3/[Total IRF3] signal was set to one. **D**. Quantification of phospho-Y701 STAT1 immunoblot signal normalized to actin; mean ± s.d., *n* = 3, *p < 0.05, unpaired two-tailed *t*-tests. For each replicate, the WT pSTAT1/actin signal was set to one. **E–I**. RT-qPCR of *IFNB1, OAS2, OAS3, ISG54*, and *ISG56* expression in the indicated cells following the transfection of 4 μg HT-DNA; mean ± s.d., *n* = 3, *p < 0.05, **p < 0.01, ***p < 0.001, ****p < 0.0001, unpaired two-tailed *t*-tests. **Alt text: A**. Bar graph comparing cGAMP concentration values across six samples: TREX1-knockout, Halo-TREX1-wildtype, two clones of Halo-TREX1-9PA, and two clones of Halo-TREX1-P61Q. **B**. Western blot showing bands for total IRF3 (55 kilodaltons), pIRF3 (55 kilodaltons), total STAT1 (about 90 kilodaltons), pSTAT1 (about 90 kilodaltons), and actin (40 kilodaltons) across six samples: TREX1-knockout, Halo-TREX1-wildtype, two clones of Halo-TREX1-9PA, and two clones of Halo-TREX1-P61Q. **C**. Bar graph comparing pIRF3-to-total-IRF3 ratio values across six samples: TREX1-knockout, Halo-TREX1-wildtype, two clones of Halo-TREX1-9PA, and two clones of Halo-TREX1-P61Q. **D**. Bar graph comparing pSTAT1-to-actin ratio values across six samples: TREX1-knockout, Halo-TREX1-wildtype, two clones of Halo-TREX1-9PA, and two clones of Halo-TREX1-P61Q. **E–I**. Bar graphs comparing mRNA relative quantification values across six samples: TREX1-knockout, Halo-TREX1-wildtype, two clones of Halo-TREX1-9PA, and two clones of Halo-TREX1-P61Q. Genes quantified: IFNB1 for E, OAS2 for F, OAS3 for G, ISG54 for H, and ISG56 for I.

We next asked whether Halo-TREX1-PPII mutations interfere with cGAS-STING regulation. cGAMP ELISA analysis following stimulation by HT-DNA transfection demonstrated significant increases in intracellular cGAMP levels in multiple, independently isolated Halo-TREX1-9PA (1.092±0.1379 s.d. fmol/μg protein for clone 1; 1.161±0.06758 s.d. fmol/μg protein for clone 2) and Halo-TREX1-P61Q (1.171±0.1046 s.d. fmol/μg protein for clone 1; 0.8806±0.1024 s.d. fmol/μg protein for clone 2) mutant cell lines relative to a wild-type Halo-TREX1 control line (0.3194±0.07516 s.d. fmol/μg protein) (Fig. 4A). Indeed, cGAMP levels in lysates prepared from Halo-TREX1-P61Q and Halo-TREX1-9PA mutant cells more closely resembled levels measured in *TREX1* KO lysates (1.374±0.04191 s.d. fmol/μg protein). cGAMP levels were unchanged in all cell lines tested, including *TREX1* KO lines, following mock transfection, further confirming that MCF10A cells lack sufficient cytosolic dsDNA to activate an immune response under baseline conditions (data not shown).

Following cGAS-STING activation, TBK1 phosphorylates the transcription factor IRF3 at multiple residues including S386 and S396, inducing IRF3 dimerization and transcription of type I IFN (29). Increased type I IFN signaling results in the phosphorylation and activation of STAT1 (pY701)/STAT2 heterodimers, ultimately culminating in the transactivation of a wide-ranging pro-inflammatory response (30). We therefore immunoblotted for phospho-IRF3 (pS386) and phospho-STAT1 (pY701) to assess cGAS-STING signaling downstream of cGAMP production (Fig. 4B-D). Consistent with prior work (22), *TREX1* KO cells exhibited significant increases in the phosphorylated forms of IRF3 and STAT1 following HT-DNA stimulation relative to wild-type Halo-TREX1 controls (Fig. 4B-D). Congruent with the observed increase in cGAMP levels, Halo-TREX1-9PA and Halo-TREX1-P61Q mutant cells exhibited increased levels of pIRF3 and pSTAT1 compared to wild-type controls, albeit to a lesser extent than *TREX1* KO cells (Fig. 4B-D).

We next performed RT-qPCR to measure *IFNB1* and associated ISG mRNA levels to test if increases in cGAMP, and IRF3, and STAT1 phosphorylation are associated with elevated pro-inflammatory gene expression. Indeed, *IFNB1, OAS2, OAS3, ISG54*, and *ISG56* transcripts were elevated across multiple Halo-TREX1-9PA and Halo-TREX1-P61Q mutant subclones relative to wild-type controls to levels that were often indistinguishable from *TREX1* KO cells (Fig. 4E-I). Taken together, these results indicate that TREX1 PPII mutations result in defective cGAS regulation and an increased pro-inflammatory transcriptional response, defects most likely stemming from TREX1 protein instability and associated reductions in overall TREX1 protein levels and corresponding decreases in nucleolytic activity.

## Discussion

Genetic associations of type I interferonopathies like AGS have been well-characterized, particularly in cases involving *TREX1* mutations linked with compromised catalytic activity (25). Yet, how missense mutations outside of the catalytic site can lead to inflammatory disease has often remained unclear. Here, we identify an AGS-linked P61Q point mutation within the non-catalytic PPII motif of TREX1. Using *in vitro* biochemical measures of protein stability and endogenous gene editing, we show that TREX1 PPII mutations, including P61Q, destabilize the protein, resulting in significantly decreased TREX1 protein levels, diminished TREX1 exonucleolytic activity, and impaired cGAS-STING regulation. These defects were obscured in lentiviral delivery models where massive overexpression of TREX1 PPII mutants masks reductions in protein stability to maintain effective cGAS inhibition. The distal position of PPII to the catalytic site, along with the lack of differences in the GFP-TREX1 lysate-based nuclease assay, suggests that the nucleolytic defect observed in the endogenous system is due to decreased protein levels, rather than a direct effect of the mutations on catalysis. Thus, our results indicate diminished protein stability and an associated reduction in overall nucleolytic power of TREX1 as a plausible molecular explanation for why TREX1 P61Q mutations lead to severe AGS phenotypes in patients.

Autoinflammatory disease-linked *TREX1* missense mutations often affect residues that play direct roles in DNA binding (i.e. R128H, K160R), catalytic activity (D18N/H, H195Y/Q, D200H/N) or dimerization (R97H, R114H). We recently reported that *TREX1* mutations may also cause dysfunction by interfering with TREX1 interactions with cGAS-DNA condensates (E198K) (19). Here, the identification of AGS-linked TREX1 P61Q mutations suggests that another class of mutations may compromise TREX1 function by diminishing overall protein stability. Indeed, structural analyses predict that the disease-linked TREX1 T13N, T32R, R185C, and D220G substitutions are likely to diminish protein stability (20). Biochemical experimentation supports this premise as TREX1 T13N, T32R, R185C, and D220G substituted proteins exhibit T_m_ reductions of 4–8 ºC *in vitro* (20). Thus, TREX1 protein destabilization may be a common defect occurring across multiple AGS-linked *TREX1* mutations.

TREX1 P61 is located with the PPII polyproline helix, a proline-rich region containing 9 prolines within a 15 amino acid stretch (23,27). This type of proline-rich segment is a conserved feature of TREX1, as it occurs in all organisms harboring TREX1, including placental mammals and marsupials. The paralog TREX2, as well as the ancient TREX nuclease occurring in non-mammals such as *Anopheles* and *Drosophila*, lack a proline-rich motif (27), indicating that PPII likely evolved during the gene duplication event. Interestingly, the emergence of PPII in evolution seems to have coincided with the addition of a long C-terminal intrinsically disordered region. In keeping with structure-based predictions based on the PPII positioning outside of the catalytic core and ER transmembrane domains, our data confirm that the PPII motif is dispensable for TREX1 nucleolytic activity and subcellular localization. The precise function of the PPII motif therefore remains unknown.

The close positioning of the two PPII helices along the same side of the TREX1 dimer interface has been proposed to create a surface that allows for protein-protein interactions without occluding the active sites (23,27). Indeed, their high potential for presenting exposed hydrogen bond donors and acceptors, cause proline-rich motifs to be considered likely protein interaction domains (31). The amino acid sequence of PPII matches the binding motif for the WW domain (27), a peptide module characterized by two tryptophan residues (32). Co-immunoprecipitation experiments have previously confirmed that murine TREX1 PPII interacts with the WW domain protein CA150 *in vitro* (27). Whether human TREX1 also interacts with WW domain proteins and endogenous interactors remains unknown. Outside of a proposed interaction with the nucleosome assembly SET protein (33), TREX1 protein partners are largely uncharacterized. Further work is therefore necessary to investigate this exciting hypothesis.

Our study relies heavily on the N-terminal HaloTag for studying the behavior of endogenous *TREX1* PPII mutations. We observed an apparent stabilizing effect of the HaloTag on TREX1, as Halo-TREX1(WT)/Δ yielded a stronger immunoblot signal than parental cells (data not shown). This observation is consistent with a prior report, which demonstrated that HaloTags can elicit a significant impact on the detection of proteins by Western blot (34). Apparent increases of HaloTag protein levels were attributed to enhanced western blot transfer efficiency (34). Therefore, western blotting analysis may underestimate the full extent of TREX1 P61Q protein instability. A further potential limitation of our study is the use of the non-malignant MCF10A breast epithelial cell line to model AGS-linked *TREX1* mutations. MCF10A cells were selected for this study because they possess an intact cGAS-STING-TREX1 pathway (22) and are suitable for facile gene editing. However, it is not clear how well this cell model recapitulates aspects of AGS, a disease that primarily affects the central nervous system. Nevertheless, orthogonal measurements of TREX1 P61Q stability via Thermofluor analysis provide assurance that the P61Q mutation is likely to exert a destabilizing effect across multiple cell types and thus reinforce our proposed mechanism of pathogenesis in patients harboring the TREX1 P61Q mutation.

## Materials and Methods

### Experimental Model and Subject Details

MCF10A cells were cultured in a 1:1 mixture of F12:DMEM media, supplemented with 5 % horse serum (Thermo Fisher Scientific #26050088), 20 ng/mL human EGF (Sigma Aldrich #E9644-.2mg), 0.5 mg/mL hydrocortisone (Sigma Aldrich), 100 ng/mL cholera toxin (Sigma Aldrich #H0888), 10 μg/mL recombinant human insulin (Sigma Aldrich #I9278-5ml), and 1% penicillin-streptomycin (Thermo Scientific #15140122). All media were supplied by the MSKCC Media Preparation core facility.

For HaloTag insertion and PPII gene editing of endogenous *TREX1* in MCF10A cells, an RNP mix was prepared by mixing 10 μg purified SpCas9 and 500 pmol of sgRNA (TREX1_gRNA#1, see Key Resources Table). After a 10-minute incubation at room temperature, 2500 ng of the pUC19-HA-Halo-TREX1 plasmid harboring the desired mutation was added to the RNP mix. The RNP-plasmid mixture was nucleofected using 4D-Nucleofector X Unit (Lonza). Fluorescence-activated cell sorting was used to isolate single-cell clones from the polyclonal cell population. For monoallelic knockout of *TREX1*, a pUC19-BBsI-CBh-TREX1_gRNA#2-Cas9-T2A-mCherry plasmid (22) was transfected using Lipofectamine 3000 (Invitrogen #L3000075). Single-cell clones were isolated by limiting dilution culture.

### Viral Transduction

For lentiviral transduction, open reading frames were cloned into pLenti-CMV-GFP-blast plasmids. Constructs were transfected into 293FT cells together with psPAX2 (Addgene #12260) and pMD2.G (Addgene #12259) using calcium phosphate precipitation. Supernatants containing lentivirus were filtered through a 0.45 μm filter and supplemented with 4 μg/mL polybrene. Successfully transduced cells were selected using 5 μg/mL blasticidin (Thermo Fisher Scientific #R21001).

### Nuclease Assay with Recombinant TREX1

*In vitro* DNA degradation assay was performed as previously described with minor modifications (20). Briefly, 1 μM 100-bp dsDNA (see below for sequence) was incubated with 0.1 μM human TREX1 or TREX1 variants in a 20 μL reaction system (20 mM Tris-HCl pH 7.5, 15 mM NaCl, 135 mM KCl, 5 mM MgCl2, and 1 mg/ml BSA) at 25°C with a time gradient of 5–30 min. DNA degradation was quenched by adding SDS (final concentration at 0.0167% (w/v)) and EDTA (final concentration at 10 mM) and incubating at 75°C for 15 min. The remaining DNA was separated on a 4% agarose gel using 0.5 × TB buffer (45 mM Tris, 45 mM boric acid) as a running buffer. After DNA electrophoresis, the agarose gel was stained with 0.5x TB buffer (containing 10 μg/mL ethidium bromide) at 25°C for 15 min, followed by de-staining with milli-Q water for an additional 45 min. DNA was visualized by ImageQuant 800 Imaging System and quantified using FIJI (35).

100-bp dsDNA sense:

5′-ACATCTAGTACATGTCTAGTCAGTATCTAGTGATTATCTAGACATACATCTAGTACATGTCTA GTCAGTATCTAGTGATTATCTAGACATGGACTCATCC -3′

100-bp dsDNA anti-sense:

5′-GGATGAGTCCATGTCTAGATAATCACTAGATACTGACTAGACATGTACTAGATGTATGTCTA GATAATCACTAGATACTGACTAGACATGTACTAGATGT -3′

### Nuclease Assay in Cell Lysates

dsDNA substrate was prepared by annealing oligo 1 (IDT; /5TEX615/GCTAGGCAG) and oligo 2 (IDT; CTGCCTAGC/3IAbRQSp/) in DNA duplex buffer (100 mM KAc, 30 mM HEPES pH 7.5) at a 1:1.15 ratio.

Whole cell lysates were generated by resuspending 3 million cells in 80 μL of assay buffer containing 25 mM HEPES 7.5, 20 mM KCl, 1 mM DTT, 1% Triton X-100, 0.25 mM EDTA, and 10 mM MgCl_2_ supplemented with Complete Mini Protease Inhibitor Cocktail (Invitrogen #11836153001). Cells were lysed by passing the cell resuspension through a 28 G syringe (BD #329461) ten times, incubated on ice for 15 minutes, and then were spun down at 14,000 × *g*, 4ºC for 15 minutes to remove pellets. 1:10 dilution of whole cell lysates in assay buffer were used to quantify protein content using Reducing Agent-compatible Pierce BCA Assay Kit (Thermo Fisher Scientific #23250).

2.5 μg (Fig. 1E) or 50 μg (Fig. 3E) of protein was loaded onto a 384-well F-bottom polystyrene microplate (Greiner Bio-One International AG Cat# 784076) with 1 μM dsDNA substrate in assay buffer. The fluorescence intensity (excitation = 570 nm, emission = 615 nm) of the plate was read immediately with Cytation 3 Multi-mode Reader (BioTek) at 25º C for 4 hours every 3 minutes.

### Live-cell Imaging

Cells were plated onto 4-well glass-bottom μ-slide dishes (Ibidi #80427) 24 h before imaging. Five minutes before imaging, media in each well was replaced with FluoroBrite DMEM Imaging Media (Thermo Scientific #A1896701) containing 1 μM ER Tracker Red (Thermo Scientific #E34250) or ER Tracker Green (Thermo Scientific #E34251). Live-cell imaging was performed at room temperature using Nikon Eclipse Ti2-E equipped with CSU-W1 SoRa spinning disk super resolution confocal system, Borealis microadapter, Perfect Focus 4, motorized turret and encoded stage, 5-line laser launch [405 (100 mw), 445 (45 mw), 488 (100 mw), 561 (80 mw), 640 (75 mw)], PRIME 95B Monochrome Digital Camera, and CFI Apo TIRF 60x 1.49 NA objective lens. Images were acquired using NIS-Elements Advanced Research Software on a Dual Xeon Imaging workstation. Adjustment of brightness and contrast were performed using Fiji software. Images were cropped and assembled into figures using Illustrator 2024 (Adobe).

### Immunoblotting

Whole cell lysates were generated by resuspending 1 million cells in RIPA buffer (25 mM Tris-HCl pH 7.6, 150 mM NaCl, 1 % NP-40, 1 % sodium deoxycholate, 0.1 % SDS) supplemented with phosphatase inhibitors (10 mM NaF, 20 mM β-glycerophosphate) and 100 μM phenylmethylsulfonyl fluoride. Cells were lysed by sonication for 15 cycles (high, 30 seconds on, 30 seconds off) using Bioruptor Plus (Diagenode). After a 15-minute incubation on ice and centrifugation (21,000 × *g*, 4 ºC for 20 minutes), pellets were removed. 1:10 dilution of whole cell lysates in RIPA were used to quantify protein content using Pierce BCA Assay Kit (Thermo Fisher Scientific #23227). 20 μg protein was loaded per sample into 15-well Novex WedgeWell Tris-Glycine Mini gels (Invitrogen #XP08165BOX). Gels were run at 120 V for 90 minutes and then transferred onto 0.45 μm nitrocellulose membranes (Cytiva #10600002) at 100 V for 60 minutes on ice. Membranes were blocked in Intercept Blocking Buffer (LI-COR #NC1660556). Primary antibodies were diluted (1:4000 for β-actin, 1:1000 for all others) in Intercept T20 (TBS) Antibody Diluent (LI-COR #927-65001) and incubated with membranes overnight at 4 ºC on a nutator. Membranes were washed three times in TBST. Secondary antibodies were diluted 1:10,000 in Intercept T20 (TBS) Antibody Diluent and incubated for 1 hour at room temperature on a shaker. After three rounds of washing with TBST and one round of washing with TBS, membranes were scanned using the Odyssey XL infrared imaging scanner (LI-COR).

### 2′3′-cGAMP Quantification

2 million cells were seeded onto 10-cm dishes 24 hours before transfection. Each plate was either transfected with 4 μg herring testes (HT-) DNA or mock-transfected using Lipofectamine 3000 (Thermo Fisher Scientific #L3000075). 24 hours after transfection, cells were harvested, washed with PBS, pelleted, flash-frozen in liquid nitrogen, and stored at –80º C. To quantify 2′3′-cGAMP levels, 2 million cells were resuspended in 200 μL LP2 lysis buffer (20 mM Tris-HCl pH 7.7, 100 mM NaCl, 10 mM NaF, 20 mM β-glycerophosphate, 5 mM MgCl_2_, 0.1 % Triton X-100, 5 % glycerol). Cells were lysed by passing the cell resuspension through a 28 G syringe (BD #329461) ten times, incubated on ice for 15 minutes, and then were spun down at 21,300 *g*, 4º C for 20 minutes to remove pellets. 2′3′-cGAMP levels were quantified using the 2′3′-cGAMP ELISA Kit (Arbor Assays #K067-H5) according to the manufacturer’s instructions. 1:10 dilution of lysates in LP2 buffer were used to quantify protein content using Pierce BCA Assay Kit (Thermo Fisher Scientific #23227). The resulting 2′3′-cGAMP levels were normalized to protein content in each sample.

### RT-qPCR

Total RNA was isolated from 1 million cells using Quick RNA Miniprep Kit (Zymo Research #R1055) according to the manufacturer’s instructions. A DNase I digestion step was included prior to eluting the RNA. cDNA was generated from 1000 ng total RNA using the SuperScript IV First-strand Synthesis System (Invitrogen #18091200) with random hexamer and oligo-(dT) priming. Reverse-transcribed samples were treated with RNase H to remove RNA. qPCR was performed with gene-specific primers (see Key Resources Table) and SYBR Green qPCR Master Mix (Applied Biosystems #A25742). qPCR was performed on QuantStudio 6 (Applied Biosystems), using 10 ng of cDNA and 250 nM of each primer on a MicroAmp 384-well reaction plate (Applied Biosystems #4309849). Relative transcription levels were calculated by normalizing to the geometric mean of *ACTB* and *GAPDH* cycle threshold values.

### Protein Expression and Purification

Large-scale protein expression was performed as previously described (36, 37). Briefly, the DNA sequences of recombinant proteins were cloned into a custom pET16 vector for expression of a 6 × His-SUMO2 fusion protein in *E. coli* BL21-RIL DE3 bacteria (Agilent) co-transformed with a pRARE2 tRNA plasmid. Starter cultures of *E. coli* were grown in MDG media, subsequently cultured in ~2 L of M9ZB media, and induced with IPTG. Bacterial cultures were pelleted, flash-frozen in liquid nitrogen, and stored at −80°C until purification.

Protein purification was performed as previously described (36, 37, 38). Briefly, bacterial pellets were re-suspended in lysis buffer (20 mM HEPES-KOH pH 7.5, 400 mM NaCl, 10% glycerol, 30 mM imidazole, 1 mM DTT) and lysed by sonication. The initial purification was performed using Ni-NTA (QIAGEN) affinity chromatography. Protein eluted from Ni-NTA was supplemented with ~250 μg of human SENP2 protease to remove the SUMO2 solubility tag and dialyzed in dialysis buffer (20 mM HEPES-KOH pH 7.5, 150 mM NaCl, 1 mM DTT) at 4°C for ~14 h. Untagged protein was further purified using Heparin HP ion-exchange (GE Healthcare) and eluted with a gradient of 150–1000 mM NaCl. Target protein was then further purified with size-exclusion chromatography using a 16/600 Superdex S75 column (GE Healthcare) equilibrated with protein storage buffer (20 mM HEPES-KOH pH 7.5, 250 mM KCl, 1 mM TCEP). The final recombinant protein was concentrated to ~20 mg/mL, flash-frozen in liquid nitrogen, and stored as aliquots at −80°C for further usage.

### Thermal Denaturation Assay

10 μM of purified TREX1 mutant protein and 3× SYPRO Orange Protein dye (Life Technologies) were loaded into a 96-well reaction plate, in a 20 μL reaction containing 20 mM Tris-HCl pH 7.5, 75 mM KCl, and 1 mM TCEP. Reactions were incubated with an increasing temperature from 20 to 95º C in a Bio-Rad CFX thermocycler with HEX channel fluorescence measurements taken every 0.5º C, and melting temperature (T_m_) was defined as the temperature at which the half of the maximum fluorescence change occurs.

### Cycloheximide Chase

2.5 million cells were seeded onto 10-cm dishes 21 hours before treatment. Each plate was treated with 7 mL of media containing 70 μg/mL cycloheximide (CHX; Sigma-Aldrich #C7698). Cells were harvested at indicated timepoints, washed with PBS, pelleted, flash-frozen in liquid nitrogen, and stored at –80 ºC. Immunoblotting was performed as indicated above.

### Statistical Analysis

Information regarding biological replicates, sample size, and statistical testing is provided in the figure legends.

## Supplementary Material

Supplementary Figure Legends

Supplementary Figures

## Figures and Tables

**Table 1:** Key Resources Table. This table reports the reagents necessary for reproducing the results presented in this manuscript.

Reagent or Resource	Source	Identifier
**Antibodies**		
GFP	Santa Cruz Biotechnology	Cat#sc-9996; RRID:AB_627695
IRF3	Abcam	Cat#ab76409; RRID:AB_1523835
pIRF3 (S386)	Abcam	Cat#ab76493; RRID:AB_1523836
pSTAT1 (Y701)	Cell Signaling Technologies	Cat#9167; RRID:AB_561284
STAT1	Cell Signaling Technologies	Cat#9176; RRID:AB_2240087
TREX1	Abcam	Cat#ab185228; RRID:AB_2885196
β-actin (mouse)	Abcam	Cat#ab8224; RRID:AB_449644
β-actin (rabbit)	Abcam	Cat#ab8227; RRID:AB_2305186
Goat anti-mouse IgG Alexa Fluor Plus 680	Invitrogen	Cat#A32729; RRID:AB_2633278
Goat anti-mouse IgG Alexa Fluor Plus 800	Invitrogen	Cat#A32730; RRID:AB_2633279
Goat anti-rabbit IgG Alexa Fluor Plus 680	Invitrogen	Cat#A32734; RRID:AB_2633283
Goat anti-rabbit IgG Alexa Fluor Plus 800	Invitrogen	Cat#A32735; RRID:AB_2633284
**Chemicals, Peptides, and Recombinant Proteins**		
Cholera Toxin	Sigma-Aldrich	Cat#C8052-2mg
cOmplete Mini Protease Inhibitor Cocktail	Sigma-Aldrich	Cat#11836153001
Cycloheximide	Sigma-Aldrich	Cat#C7698
ER Tracker Green	Invitrogen	Cat#E34251
ER Tracker Red	Invitrogen	Cat#E34250
Horse Serum	Thermo Fisher Scientific	Cat#26050088
Human EGF	Sigma-Aldrich	Cat#E9644-.2mg
Hydrocortisone	Sigma-Aldrich	Cat#H0888
Insulin	Sigma-Aldrich	Cat#I9278-5ml
Penicillin-Streptomycin (10,000 U/mL)	Thermo Fisher Scientific	Cat#15140122
FluoroBrite DMEM	Thermo Fisher Scientific	Cat#A1896701
Janelia Fluor HaloTag Ligand 646	Promega	Cat#GA1120
ER Tracker Green	Thermo Fisher Scientific	Cat#E34251
ER Tracker Red	Thermo Fisher Scientific	Cat#E34250
Lipofectamine 3000	Thermo Fisher Scientific	Cat#L3000075
Blasticidin S HCl, powder	Thermo Fisher Scientific	Cat#R21001
Amersham Protran 0.45 NC Nitrocellulose Membrane	Cytiva	Cat#10600002
Novex WedgeWell Tris Glycine Mini gels	Invitrogen	Cat#XP08165BOX
Intercept T20 (TBS) Antibody Diluent	LI-COR	Cat#927-65001
Intercept Blocking Buffer	LI-COR	Cat#NC1660556
Quick-RNA Miniprep Kit	Zymo Research	Cat#R1055
SYBR Green Master Mix	Applied Biosystems	Cat#A25742
SuperScript IV First-Strand Synthesis System	Invitrogen	Cat#18091200
**Critical Commercial Assays**		
2′3′-Cyclic GAMP Direct EIA Kit	Arbor Assays	Cat#K067-H5
Pierce BCA Protein Assay Kit	Thermo Fisher Scientific	Cat#23227
Pierce BCA Protein Assay Kit, Reducing Agent-compatible	Thermo Fisher Scientific	Cat#23250
**Experimental Models: Cell Lines**		
MCF10A	Maria Jasin Lab	N/A
MCF10A TREX1-KO	this paper	cJM14
MCF10A TREX1-KO + GFP-TREX1	this paper	cAS1
MCF10A TREX1-KO + GFP-TREX1(8PA)	this paper	cAS2
MCF10A TREX1-KO + GFP-TREX1(P61Q)	this paper	cAS3
MCF10A Halo-TREX1(WT/Δ)	this paper	cAS4
MCF10A Halo-TREX1(9PA/Δ)	this paper	cAS5
MCF10A Halo-TREX1(P61Q/Δ)	this paper	cAS6
**Oligonucleotides**		
ACTB F for qPCR (ATCTGGCACCACACCTTCTAC)	this paper	N/A
ACTB R for qPCR (CAGCCAGGTCCAGACGCAGG)	this paper	N/A
GAPDH F for qPCR (CATCACCATCTTCCAGGAGCGA)	this paper	N/A
GAPDH R for qPCR (CCTGCTTCACCACCTTCT)	this paper	N/A
IFNB1 F for qPCR (TACTGCCTCAAGGACAGGATGAA)	Li et al., 2023 (PMID: 37612508)	N/A
IFNB1 R for qPCR (GCATCTCATAGATGGTCAATGCG)	Li et al., 2023 (PMID: 37612508)	N/A
OAS2 F for qPCR (GAGCCAGTTGCAGAAAACCAG)	Bakhoum et al., 2018 (PMID: 29342134)	N/A
OAS2 R for qPCR (GCATTGTCGGCACTTTCCAA)	Bakhoum et al., 2018 (PMID: 29342134)	N/A
OAS3 F for qPCR (GAAGCCCAGGCCTATCATCC)	Bakhoum et al., 2018 (PMID: 29342134)	N/A
OAS3 R for qPCR (TCATCCAGTAGGACCGCTGA)	Bakhoum et al., 2018 (PMID: 29342134)	N/A
ISG54 F for qPCR (ACGGTATGCTTGGAACGATTG)	Diner et al., 2015 (PMID: 25693804)	N/A
ISG54 R for qPCR (AACCCAGAGTGTGGCTGATG)	Diner et al., 2015 (PMID: 25693804)	N/A
ISG56 F for qPCR (AAGGCAGGCTGTCCGCTTA)	Diner et al., 2015 (PMID: 25693804)	N/A
ISG56 R for qPCR (TCCTGTCCTTCATCCTGAAGCT)	Diner et al., 2015 (PMID: 25693804)	N/A
TREX1 F for qPCR (GCATCTGTCAGTGGAGACCA)	Yan et al., 2010 (PMID: 20871604)	N/A
TREX1 R for qPCR (AGATCCTTGGTACCCCTGCT)	Yan et al., 2010 (PMID: 20871604)	N/A
Oligo 1 for nuclease activity assay (/5TEX615/GCTAGGCAG)	this paper	N/A
Oligo 2 for nuclease activity assay (CTGCCTAGC/3IAbRQSp/)	this paper	N/A
TREX1; guide RNA #1 (GCAGGTACGTACCCAACCAT)	Umbreit et al., 2020 (PMID: 32299917)	N/A
TREX1; guide RNA #2 (GAGCCCCCCCACCTCTC)	this paper	N/A
**Recombinant DNA**		
pLenti-CMV-GFP-TREX1-BLAST	Mohr et al., 2021 (PMID: 33476576)	Addgene #164228
pLenti-CMV-GFP-TREX1(8PA)-BLAST	this paper	
pLenti-CMV-GFP-TREX1(P61Q)-BLAST	this paper	
pUC19-HA-Halo-TREX1(9PA)	this paper	
pUC19-HA-Halo-TREX1(P61Q)	this paper	
psPAX2	gift from Didier Trono	Addgene #12260
pMD2.G	gift from Didier Trono	Addgene #12259

## Abbreviations

8PA: eight proline-to-alanine mutations
9PA: nine proline-to-alanine mutations
AGS: Aicardi-Goutières syndrome
ANOVA: analysis of variance
cGAMP: 2′3′-cyclic GMP-AMP
cGAS: cyclic GMP-AMP synthase
CHX: cycloheximide
CMV: cytomegalovirus
CRISPR: clustered regularly interspaced short palindromic repeats
DAPI: 4’,6-diamidino-2-phenylindole
DMEM: Dulbecco’s Modified Eagle Medium
DNA: deoxyribonucleic acid
dsDNA: double-stranded deoxyribonucleic acid
DTT: dithiothreitol
EDTA: ethylenediaminetetraacetic acid
ELISA: enzyme-linked immunosorbent assay
ER: endoplasmic reticulum
GFP: green fluorescent protein
HCl: hydrochloric acid
HT-DNA: herring testes deoxyribonucleic acid
KCl: potassium chloride
IFN: interferon
ISG: interferon-stimulated gene
KO: knockout
MgCl_2_: magnesium chloride
mRNA: messenger ribonucleic acid
NaCl: sodium chloride
NaF: sodium fluoride
NIS: Nikon Imaging Software
P61Q: proline-61-to-glutamine mutation
PBS: phosphate-buffered saline
PCR: polymerase chain reaction
PPII: polyproline II helix
RIPA: radioimmunoprecipitation assay buffer
RNP: ribonucleoprotein
RT-qPCR: reverse transcription-quantitative polymerase chain reaction
RVCL: retinal vasculopathy with cerebral leukoencephalopathy
SDS: sodium dodecyl sulfate
SLE: systemic lupus erythematosus
STING: stimulator of interferon genes
TB: tris/borate
TBS: tris-buffered saline
TBST: tris-buffered saline with Tween 20
TCEP: tris(2-carboxyethyl)phosphine
T_m_: melting temperature
TREX1: three prime repair exonuclease 1
WT: wildtype
