# Supplementary material for: Mutations in the non-catalytic polyproline motif destabilize TREX1 and amplify cGAS-STING signaling
Source: Hum Mol Genet. Author manuscript; Available in PMC 2024 Nov 10. (PMC11373327; doi:10.1093/hmg/ddae089)
Supplement: Supplementary Figure Legends [file EMS199867-supplement-Supplementary_Figure_Legends.docx]

**HMG-2024-CE-00107**

**LEGENDS TO SUPPLEMENTARY FIGURES**

**Figure S1. Overexpressed TREX1 mutants localize to the ER.** **A.** Representative immunoblot of MCF10A *TREX1* KO cells reconstituted with the indicated GFP-TREX1, using anti-TREX1, anti-GFP, and anti-actin antibodies. **B.** Quantification of TREX1 immunoblot signal normalized to actin; mean ± s.d., *n* = 3, ns = not significant, one-way ANOVA (*p* = 0.0401). For each replicate, the parental TREX1/Actin signal was set to one. **C.** Quantification of GFP-TREX1 signal in the indicated cells as in Fig. 1G; mean ± s.d., *n* = 5 experiments, *****p* < 0.0001, one-way ANOVA (*p* < 0.0001).**D.** Line profile analysis of the indicated cells as in Fig. 1G. Position of the nucleus was determined using the line profile signal of the DAPI channel. Background signal was subtracted from all points.

**Alt-text:** **A.** Western blot showing bands for TREX1 (about 35 and 60 kilodaltons), GFP (about 60 kilodaltons), and actin (40 kilodaltons) across five genotypes: TREX1-knockout, parental, GFP-TREX1-wildtype, GFP-TREX1-8PA, and GFP-TREX1-P61Q. **B.** Bar graph comparing TREX1-to-actin ratio values across five genotypes: TREX1-knockout, parental, GFP-TREX1-wildtype, GFP-TREX1-8PA, and GFP-TREX1-P61Q. **C.** Bar graph comparing GFP-TREX1 intensity values minus background across four genotypes: parental, GFP-TREX1-wildtype, GFP-TREX1-8PA, and GFP-TREX1-P61Q. **D.** Panel of three line graphs, each representing a distinct genotype: GFP-TREX1-wildtype, GFP-TREX1-8PA, and GFP-TREX1-P61Q. Each line graph compares fluorescence intensity values of GFP and ER Tracker as a function of distance.

**Figure S2. Generation of *TREX1* knock-in mutations.** **A.** Representative schematic of *TREX1* gene editing protocol. Briefly, an N-terminal sgRNA and a HaloTag donor plasmid harboring a PPII edit in its downstream homology arm were nucleofected into MCF10A cells. Sanger sequencing revealed that HaloTag insertion occurs more frequently than incorporation of the PPII edit, often yielding two Halo-tagged alleles, one with the desired PPII edit. A second round of gene editing was carried out using a sgRNA specific for unedited PPII, knocking out the unedited allele while leaving the PPII-edited, Halo-tagged allele intact. **B.** Schematic of PCR primers and amplicons used to validate knock-in cell lines. **C.** PCR gel of all knock-in cell line clones used in this manuscript. All bands were excised and Sanger sequenced to validate expected gene edits. **D.** Schematic detailing the precise edits present in all clones.

**Alt text: A.** Step-by-step schematic of how endogenous TREX1 gene was edited using CRISPR/Cas9. **B.** Schematic of where PCR primers and amplicons are located with respect to the endogenous TREX1 locus. **C.** DNA gel showing PCR bands for six samples: parental, Halo-TREX1-wildtype, two clones of Halo-TREX1-9PA, and two clones of Halo-TREX1-P61Q. **D.** Panel of endogenous TREX1 locus schematics, showing the edits and indels made in five samples: Halo-TREX1-wildtype, two clones of Halo-TREX1-9PA, and two clones of Halo-TREX1-P61Q.

**Figure S3. Mutations in PPII do not interfere with TREX1 transcription or localization.** **A.** Pearson correlation coefficients of the indicated cells as in Fig. 3C; mean ± s.d., *n* = 5 experiments, *****p* < 0.0001, ns = not significant, two-way ANOVA (interaction *p* < 0.0001, comparison pair *p* < 0.0001, genotype *p* < 0.0001). **B.** Line profile analysis as indicated in Fig. 3C. Extent of the nucleus was determined using the line profile signal of the DAPI channel. Background signal was subtracted from all points. **C.** RT-qPCR of *TREX1* in the indicated cells following mock transfection; mean ± s.d., *n* = 3, **p* < 0.05, ***p* < 0.01, ****p* < 0.001, ns = not significant, one-way ANOVA (*p* < 0.0001). **D.** SDS-PAGE and Coomassie analysis of purified TREX1 proteins used in this study. **E.** Immunoblot of MCF10A knock-in (KI) cell lines treated with cycloheximide (CHX) for the indicated duration, using anti-TREX1 and anti-actin antibodies. **F.** Quantification of TREX1 immunoblot signal as in (D); *n* = 1. TREX1/Actin signals were normalized to the TREX1/Actin signal at t = 0h (untreated) within each genotype.

**Alt text: A.** Bar graph comparing Pearson correlation coefficient values between GFP and ER channels and between GFP and DAPI channels, across three genotypes: Halo-TREX1-wildtype, Halo-TREX1-9PA, and Halo-TREX1-P61Q. **B.** Panel of three line graphs, each representing a distinct genotype: Halo-TREX1-wildtype, Halo-TREX1-9PA, and Halo-TREX1-P61Q. Each line graph compares fluorescence intensity values of HaloTag and ER Tracker as a function of distance. **C.** Bar graph comparing TREX1 mRNA relative quantification values across five samples: Halo-TREX1-wildtype, two clones of Halo-TREX1-9PA, and two clones of Halo-TREX1-P61Q. **D.** SDS-PAGE gel showing bands for TREX1 wildtype, 9PA, and P61Q protomers. **E.** Two Western blots showing bands for TREX1 (about 70 kilodaltons) and actin (40 kilodaltons). Top Western blot includes samples treated with cycloheximide for 0, 1, 2, 4, 6, 8, or 24 hours, across two genotypes: Halo-TREX1-wildtype and Halo-TREX1-9PA. Bottom Western blot includes samples treated with cycloheximide for 0, 1, 2, 4, 6, 8, or 24 hours, across two genotypes: Halo-TREX1-wildtype and Halo-TREX1-P61Q. **F.** Two side-by-side line graphs showing decrease in TREX1-to-actin ratio values across time. Left line graph includes Halo-TREX1-wildtype and Halo-TREX1-9PA. Right line graph includes Halo-TREX1-wildtype and Halo-TREX1-P61Q.
