# Supplementary figures and images for: Mutations in the non-catalytic polyproline motif destabilize TREX1 and amplify cGAS-STING signaling
Source: Hum Mol Genet. Author manuscript; Available in PMC 2024 Nov 10. (PMC11373327; doi:10.1093/hmg/ddae089)

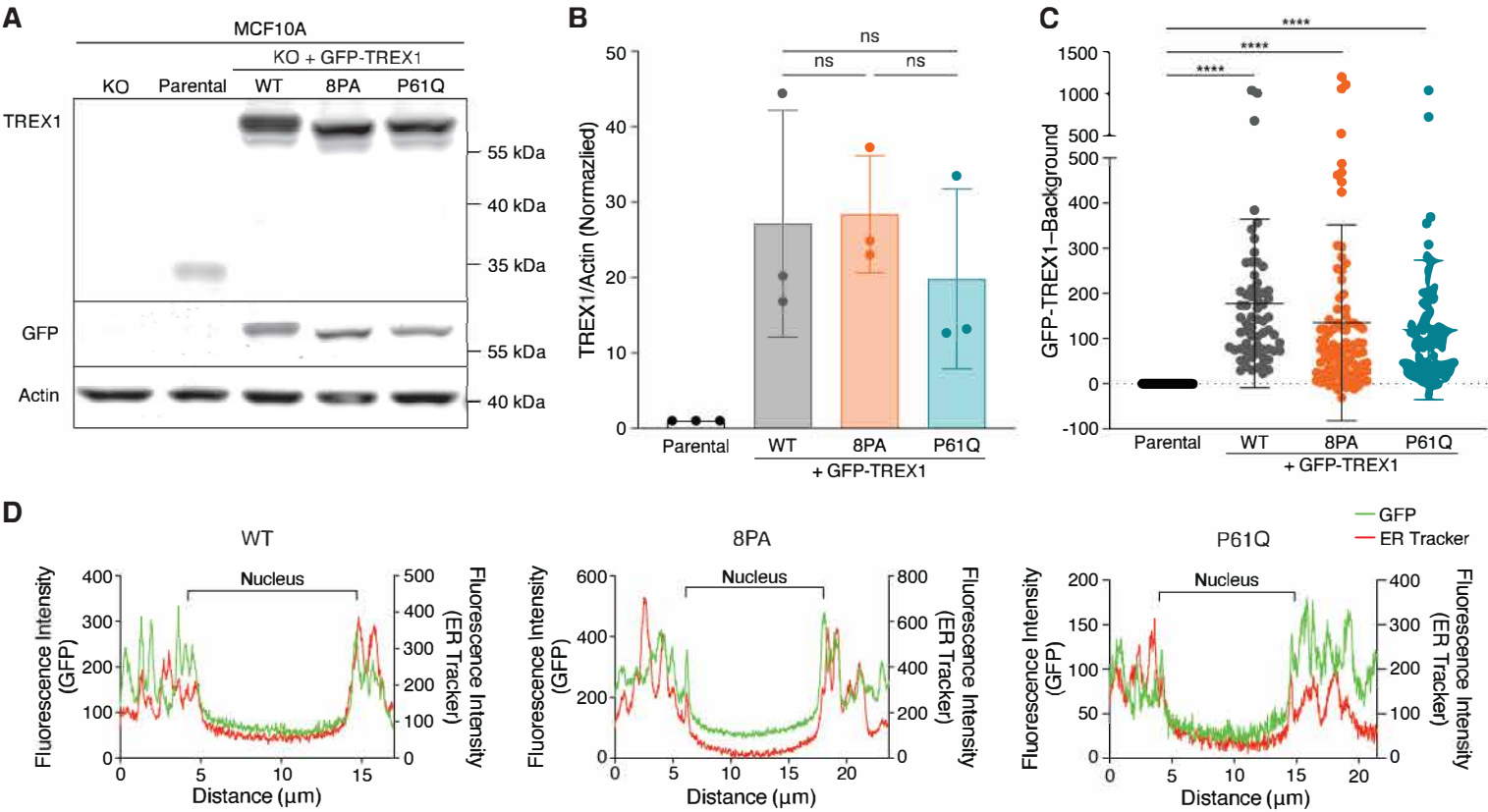

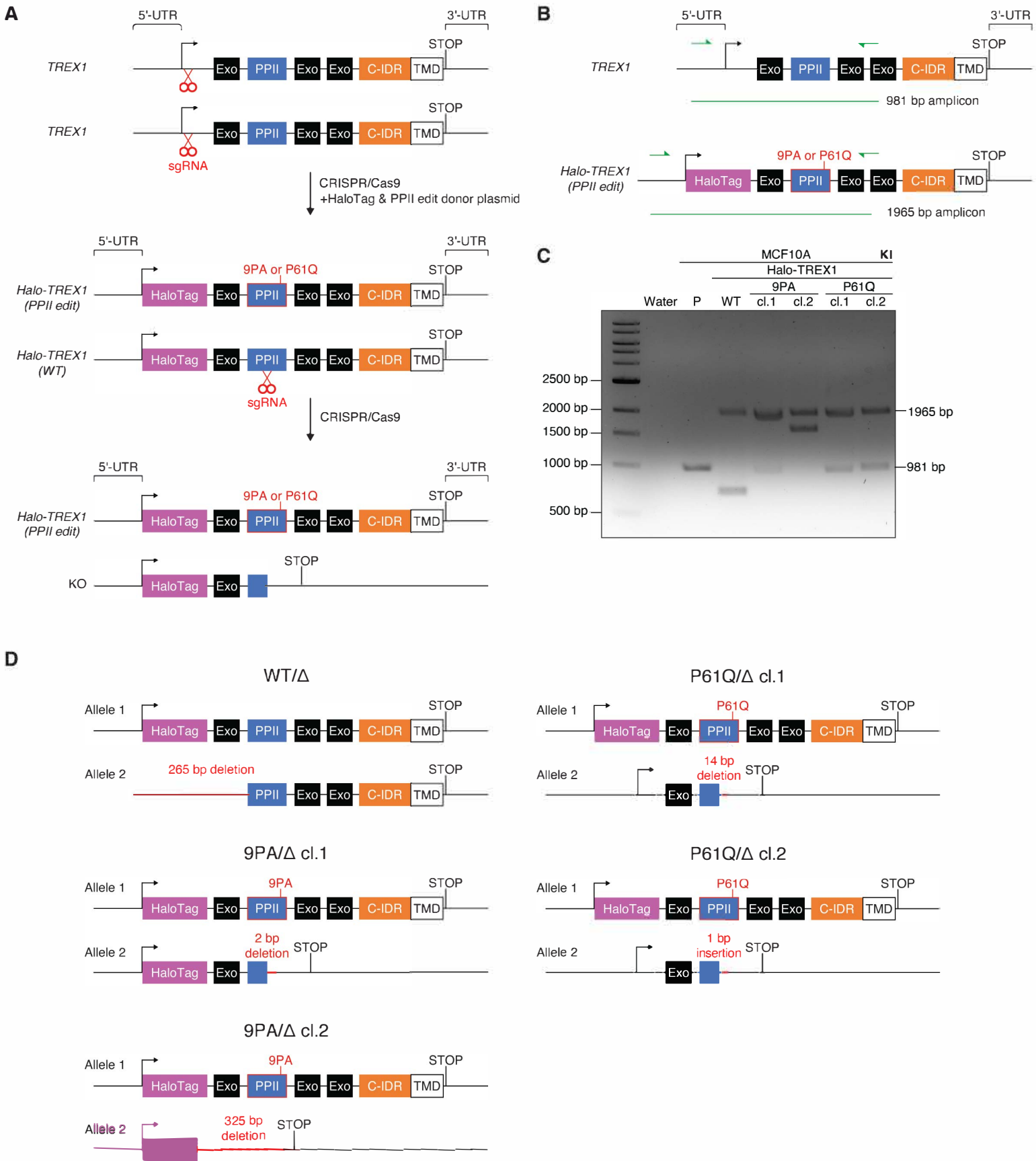

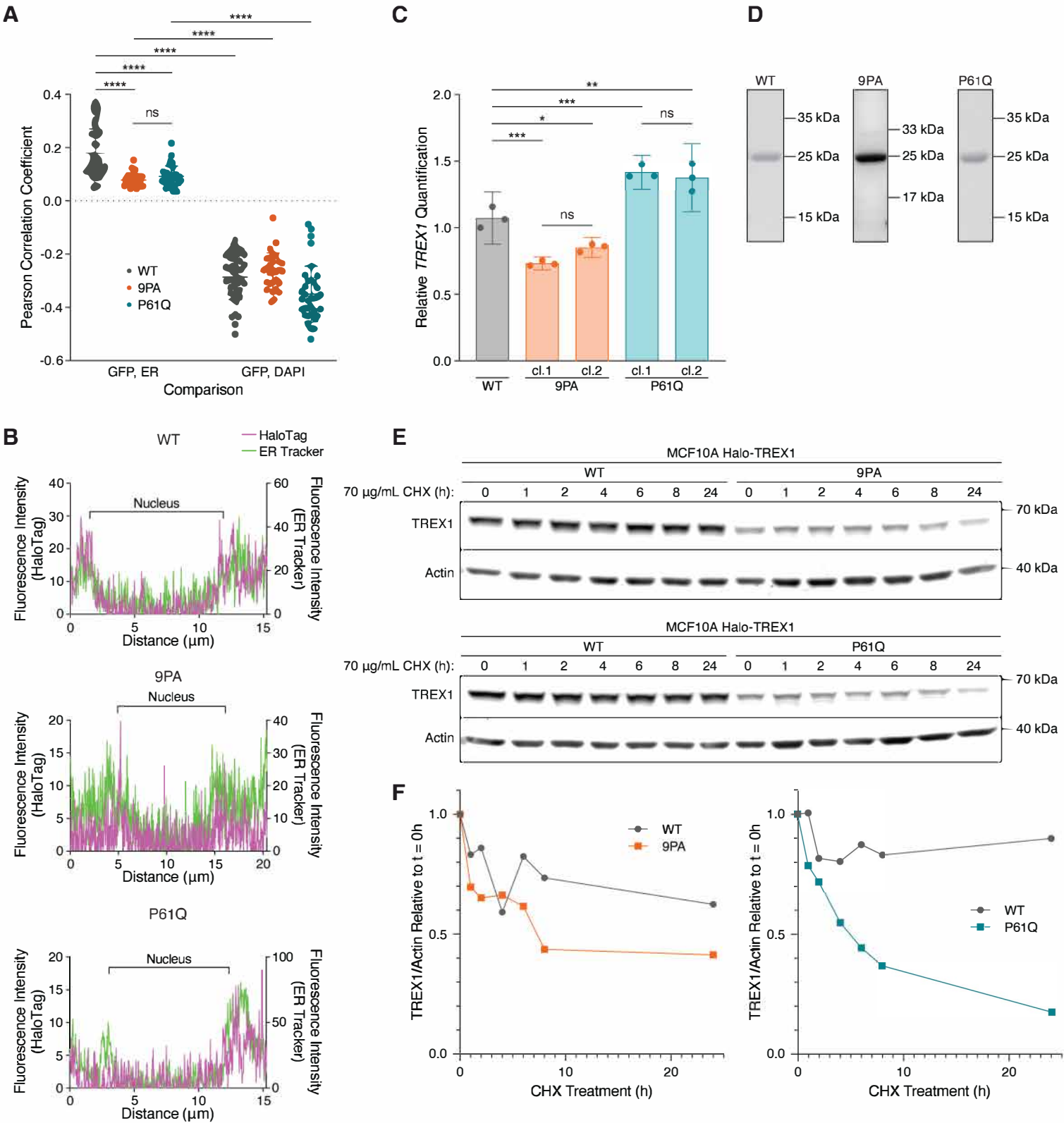

Supplement: Supplementary Figures [file EMS199867-supplement-Supplementary_Figures.pdf]
